# 17-allyamino-17-demethoxygeldanamycin treatment results in a magnetic resonance spectroscopy-detectable elevation in choline-containing metabolites associated with increased expression of choline transporter SLC44A1 and phospholipase A2

**DOI:** 10.1186/bcr2729

**Published:** 2010-10-14

**Authors:** Alissa H Brandes, Christopher S Ward, Sabrina M Ronen

**Affiliations:** 1 Department of Radiology and Biomedical Imaging, University of California, San Francisco, 1700 4th Street, San Francisco, CA 94158, USA

## Abstract

**Introduction:**

17-allyamino-17-demethoxygeldanamycin (17-AAG), a small molecule inhibitor of Hsp90, is currently in clinical trials in breast cancer. However, 17-AAG treatment often results in inhibition of tumor growth rather than shrinkage, making detection of response a challenge. Magnetic resonance spectroscopy (MRS) and spectroscopic imaging (MRSI) are noninvasive imaging methods than can be used to monitor metabolic biomarkers of drug-target modulation. This study set out to examine the MRS-detectable metabolic consequences of Hsp90 inhibition in a breast cancer model.

**Methods:**

MCF-7 breast cancer cells were investigated, and MRS studies were performed both on live cells and on cell extracts. ^31^P and ^1^H MRS were used to determine total cellular metabolite concentrations and ^13^C MRS was used to probe the metabolism of [1,2-^13^C]-choline. To explain the MRS metabolic findings, microarray and RT-PCR were used to analyze gene expression, and *in vitro *activity assays were performed to determine changes in enzymatic activity following 17-AAG treatment.

**Results:**

Treatment of MCF-7 cells with 17-AAG for 48 hours caused a significant increase in intracellular levels of choline (to 266 ± 18% of control, *P *= 0.05) and phosphocholine (PC; to 181 ± 10% of control, *P *= 0.001) associated with an increase in expression of choline transporter SLC44A1 and an elevation in the *de novo *synthesis of PC. We also detected an increase in intracellular levels of glycerophosphocholine (GPC; to 176 ± 38% of control, *P *= 0.03) associated with an increase in PLA2 expression and activity.

**Conclusions:**

This study determined that in the MCF-7 breast cancer model inhibition of Hsp90 by 17-AAG results in a significant MRS-detectable increase in choline, PC and GPC, which is likely due to an increase in choline transport into the cell and phospholipase activation. ^1^H MRSI can be used in the clinical setting to detect levels of total choline-containing metabolite (t-Cho, composed of intracellular choline, PC and GPC). As Hsp90 inhibitors enter routine clinical use, t-Cho could thus provide an easily detectable, noninvasive metabolic biomarker of Hsp90 inhibition in breast cancer patients.

## Introduction

Recent reports from the American Cancer Society indicate that nearly 1 in 8 American women will be diagnosed with breast cancer in her lifetime. As the prevalence of this disease persists, the pressure to develop novel and improved therapies increases. Cancer research has become increasingly focused on the potential of various small molecule inhibitors to target specific proteins in signaling pathways that are commonly overexpressed in cancer. Whereas these drugs have had some success in clinical trials, they still have limited efficacy in treating many cancer types and this has been largely due to cross-talk between different pathways, feedback loops, and parallel signaling [1].

This challenge might be overcome by simultaneously inhibiting several oncogenic pathways; 17-allyamino-17-demethoxygeldanamycin (17-AAG), a small molecule inhibitor of heat shock protein 90 (Hsp90), accomplishes this. Hsp90 is a molecular chaperone responsible for the folding and stability of multiple client proteins, many of which are oncogenes, including Akt, c-Raf, and Her-2 [2]. When 17-AAG inhibits Hsp90, its client proteins are degraded via the proteasome ubiquinitation pathway, resulting in inhibition of oncogenic signaling. High expression of Hsp90 has also been associated with decreased survival in breast cancer [3]. Hsp90 inhibitors, including 17-AAG, are currently undergoing clinical trials and show promise as effective anti-cancer therapies for the treatment of many cancer types, including breast cancer [4,5]. However, the treatment of tumors with 17-AAG most often results in an inhibition of tumor growth rather than shrinkage [6-8]. As a result, early response to this type of treatment is nearly impossible to detect by traditional imaging, such as computed tomography and magnetic resonance (MR) imaging. As this therapy enters routine clinical use, it would be beneficial to have a noninvasive functional or metabolic biomarker that could provide information about the molecular effects of Hsp90 inhibition in patients.

Magnetic resonance spectroscopy (MRS)-detectable phosphocholine (PC) could potentially serve as such a biomarker. Concentrations of PC and, in some cases, GPC are elevated in tumors as compared with normal tissue and have been shown to increase with increasing malignancy in breast cancer models and patient studies [9-15]. Accordingly, PC and total choline-containing metabolites (t-Cho) (composed of choline, PC, and glycerophosphocholine [GPC] and detectable by ^1^H magnetic resonance spectroscopic imaging [MRSI]) are now recognized as clinically relevant metabolic biomarkers of breast cancer [14,16,17]. Additionally, ^31^P and ^1^H MRS studies have demonstrated that PC and t-Cho levels decrease in response to chemotherapy as well as targeted therapies, both *in vitro *and *in vivo *[18-28].

However, a previous MRS-based study examining the effects of 17-AAG in a colon cancer model reported the somewhat atypical effect of increases in PC and GPC concentrations following response to treatment [29]. This finding was supported by a different positron emission tomography (PET)-based study, which reported an increase in uptake of the PET tracer [^11^C]-choline after treatment with Hsp90 inhibitor NVP-AUY922 in three different cell lines (MCF-7 human breast cancer, U87MG human glioblastoma, and HCT116 human colon cancer) [30]. GPC levels have been shown to increase in response to other anti-cancer drug treatments [31-33].

**Figure 1:**
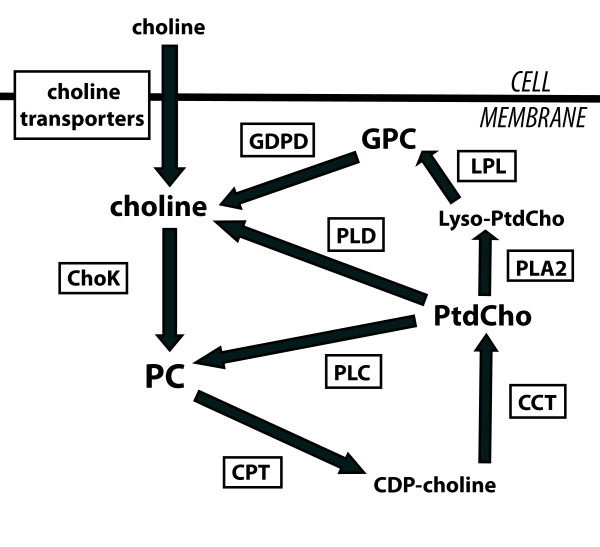
**Schematic of choline phospholipid metabolism**. Schematic drawing of choline phospholipid metabolism and its regulatory enzymes illustrates the metabolic reactions associated with modulation of choline-containing metabolites. CCT, CTP:phosphocholine cytidylyltransferase; CDP-choline, cytidine diphosphate-choline; ChoK, choline kinase; CPT, diacylglycerol cholinephosphotransferase; GDPD, glycerophosphocholine phosphodiesterase; GPC, glycerophosphocholine; LPL, lysophospholipase; PC, phosphocholine; PLA, phospholipase A; PLC, phospholipase C; PLD, phospholipase D; PtdCho, phosphatidylcholine.

The concentration of PC in the cell is regulated by the enzymes of the choline phospholipid metabolic pathway (Figure 1). Extracellular choline is actively transported into the cell by several choline transporters and subsequently is phosphorylated to PC by choline kinase (ChoK) [34,35]. CTP:phosphocholine cytidylyltransferase (CCT) metabolizes PC to cytidine diphosphate-choline (CDP-choline). Diacylglycerol cholinephosphotransferase (CPT) further metabolizes CDP-choline to one of the main membrane phospholipids, phosphatidylcholine (PtdCho), and CCT is considered the rate-limiting step for PtdCho synthesis [36]. Various phospholipase enzymes can break down PtdCho: phospholipases A1 and A2 (PLA1 and PLA2) generate fatty acids and lyso-phosphatidylcholine (Lyso-PtdCho), which is further metabolized by lysophospholipase into fatty acids and GPC; phospholipase C (PLC) generates PC and diacylglycerol; and phospholipase D (PLD) generates choline and phospahtidic acid. GPC can be further metabolized by glycerophosphocholine phosphodiesterase (GDPD) into choline. Examination of this pathway reveals three sources that could be responsible for increased PC following 17-AAG treatment: an increase in the *de novo *synthesis of PC from extracellular choline, a decrease in the further metabolism of PC via CCT, or an increase in the breakdown of PtdCho.

Since 17-AAG is currently in clinical trials in patients with breast cancer [4,5], we set out to determine whether PC and t-Cho can serve as noninvasive biomarkers of Hsp90 inhibition in this group of patients. Our goals were, first, to confirm that treatment with 17-AAG results in increases in PC and t-Cho and, second, to investigate the underlying mechanism of their modulation. We studied the effect of 17-AAG treatment on the MCF-7 breast cancer cell line by probing choline metabolism by means of MRS and by using complementary assays to determine the activity and expression of the enzymes involved in choline phospholipid metabolism. We found that 17-AAG caused increases in the intracellular choline and PC concentrations, likely resulting from increased expression of the choline transporter SLC44A1 and elevation in the *de novo *synthesis of PC. We also found that GPC levels were elevated with 17-AAG treatment, resulting from increases in gene expression and activity of PLA2.

## Materials and methods

### Cell culture

MCF-7 cells were obtained from the American Type Culture Collection (Manassas, VA, USA) via UCSF Cell Culture Facility (San Francisco, CA, USA). Cells were cultured in Dulbecco's modified Eagle's medium (DMEM) supplemented with 10% heat-inactivated fetal bovine serum, 2 mM L-glutamine, 100 units/mL penicillin, and 100 μg/mL streptomycin (UCSF Cell Culure Facility). Custom-made DMEM with 0.22 g/L inorganic phosphate (UCSF Cell Culture Facility) was used for all MRS experiments. For inhibition of Hsp90, 3 μM 17-AAG (LC Laboratories, Woburn, MA, USA) was added to the culture medium for a period of 48 hours. Medium was replenished with fresh 17-AAG every 24 hours during treatment. The doubling time of logarithmic-phase MCF-7 cells growing in tissue culture flasks or on beads was determined by seeding at a density of 1 × 10^6 ^cells per 75-cm^2 ^flask or at a density of 3 × 10^6 ^cells per 0.5 mL of beads, trypsinizing at 24-hour intervals and counting the number of cells by using a hemocytometer.

### Cell proliferation assay

The effect of 17-AAG treatment on cell proliferation was determined by using the WST-1 cell proliferation assay (Roche, Indianapolis, IN, USA). Cells were seeded in 96-well microplates (1.5 × 10^6 ^cells per well in 150 μL of culture medium), and control and 17-AAG-treated cells were probed at different time periods between 4 and 48 hours. For this, cells were incubated for 1 hour with WST-1 reagent, and cell viability was determined by quantification of the absorbance at 440 nm by using a plate reader spectrophotometer (Tecan, Grödig, Austria).

### Protein determination

Protein concentrations per cell were determined in cells lysed in Cell Lysis Buffer (Cell Signaling Technology, Danvers, MA, USA) by using a Coomassie Plus-The Better Bradford™ Assay Kit (Pierce, Rockford, IL, USA), and bovine serum albumim was used as a protein standard. Absorbance was measured at 595 nm by using a plate reader spectrophotometer (Tecan).

### Western blotting

The effect of 17-AAG on levels of client proteins was analyzed by Western blotting. Cellular lysates were extracted in Cell Lysis Buffer (Cell Signaling Technology) with added Protease Inhibitor (Calbiochem, now part of EMD Biosciences, Inc., San Diego, CA, USA). Protein concentration was determined by using a Coomassie Plus Protein Assay (Pierce). Approximately 30 μg of protein was loaded into 4% to 20% SDS-PAGE gels (Bio-Rad Laboratories, Hercules, CA, USA). Gels were run for 30 minutes and then proteins were electrotransferred onto nitrocellulose membranes. Membranes were blocked for 1 hour in 5% milk in TBST (Tris-buffered saline Tween 20) and then incubated overnight with primary antibodies against Akt, c-Raf, and Actin (Cell Signaling Technology), and Actin was used as a loading control. After incubation for 2 hours with secondary antibody anti-IgG horseradish peroxidase-linked antibody (Cell Signaling Technology), the immunocomplexes were visualized with ECL Western Blotting Substrate (Pierce).

### Flow cytometry

Cells (1 × 10^6^) were seeded in 75-cm^2 ^tissue culture flasks in 10 mL of culture medium. Following treatment, approximately 2 × 10^6 ^cells were harvested by trypsinization, washed with phosphate-buffered saline (PBS), and fixed in 70% ethanol. Fixed cells were incubated with RNase A at 100 units/mL (Sigma-Aldrich, St. Louis, MO, USA) for 30 minutes, washed with PBS, and then stained with propidium iodide at 20 μg/mL (Sigma-Aldrich) for 30 minutes. Cells were counted with a fluorescence-activated cell sorting (FACS) Calibur flow cytometer and CellQuest Pro software (BD Biosciences, Mountain View, CA, USA) and single cells were gated away from clumped cells by using forward light scattering on an FL2-width versus FL2-area dot plot. The percentages of cells in the G_1_, S, and G_2_/M phases were determined by plotting a histogram of FL2-A. The relative cell sizes of the G_1_, S, and G_2_/M phase populations of control and 17-AAG-treated cells were determined by using forward scattering height (FSC-H).

### Perfused cell magnetic resonance spectroscopy studies

Approximately 4 × 10^7 ^MCF-7 cells were grown on Biosilon microcarrier beads (Nunc, Rochester, NY, USA) in order to be loaded into a perfusion system for MRS studies as described previously [37]. Cells were seeded on the microcarrier beads, allowed to attach for 24 hours, and then treated for 48 hours prior to the MR experiment. To monitor a similar number of cells during MRS acquisition, control cells were seeded at a density of approximately 5 × 10^6 ^cells/mL of beads and 17-AAG-treated cells were seeded at a density of approximately 9 × 10^6 ^cells/mL of beads. To obtain cell counts for both the start and end of the experiment, an additional set of cells was seeded on beads for each experiment and treated in a fashion identical to those loaded into the perfusion system.

MRS studies were performed on a 500-MHz INOVA spectrometer (Varian, Santa Clara, CA, USA). Composite pulse proton-decoupled ^31^P spectra were first acquired to confirm cell viability and quantify metabolite levels. ^31^P spectra were acquired with a 30° pulse and 3-second relaxation delay and 1,000 transients for a total acquisition time of 70 minutes. Cells were then perfused with fresh culture medium containing only 56 μM [1,2-^13^C]-choline (Cambridge Isotope Laboratories, Andover, MA, USA) and no unlabeled choline for a period of 14 hours. Proton-decoupled (Waltz 16) ^13^C spectra were acquired during that time in 2-hour intervals by using a 60° pulse and 6-second relaxation delay and 1,024 transients. Spectra were quantified with ACD/Spec Manager version 9.15 software (Advanced Chemistry Development, Toronto, ON, Canada). Peak integrals obtained by deconvolution were normalized to cell number and to a metabolite of known concentration in the culture medium (1.87 μM inorganic phosphate [P_i_] for ^31^P spectra and 5 mM [1-^13^C]-glucose [Cambridge Isotope Laboratories] for ^13^C spectra). Data were corrected for saturation effects by using correction factors obtained from a fully relaxed spectrum of perfused cells (fully relaxed ^13^C spectra were acquired with a 90°, 20-microsecond pulse and 30-second relaxation delay for 1,024 transients, and fully relaxed ^31^P spectra were acquired with a 90°, 22-microsecond pulse and 30-second relaxation delay for 1,000 transients).

### Cell extracts

Cells were grown in culture with medium in which all glucose and choline were completely replaced by 5 mM [1-^13^C]-glucose and 56 μM [1,2-^13^C]-choline (Cambridge Isotope Laboratories) for a period of 48 hours prior to extraction. Approximately 7 × 10^7 ^cells were extracted by means of the dual-phase extraction method, as described previously [26,38,39]. Briefly, cultured cells were collected by trypsinization, counted to obtain cell number, and fixed in 10 mL of methanol. Chloroform (10 mL) was added and then water (10 mL) was as well. The solution was vortexed and centrifuged to separate the lipid and aqueous phases and then each phase was collected separately. Solvents were lyophilized off each sample. Aqueous samples were re-dissolved in a volume of 400 μL of deuterium oxide, and lipid samples were re-dissolved in 500 μL of deuterated chloroform-methanol mixture.

^13^C, ^1^H, and ^31^P spectra were acquired on the lipid and aqueous portions of the cell extracts by using a 600-MHz INOVA spectrometer (Varian) equipped with a 5-mm broadband observe probe. Proton-decoupled (Waltz 16) ^13^C and ^31^P spectra were acquired with a 30° pulse and 3-second relaxation delay. ^13^C aqueous spectra were acquired for 5,000 transients and a total acquisition time of 6 hours. ^13^C lipid spectra were acquired for 2,500 transients and a total acquisition time of 3 hours. ^31^P aqueous and lipid spectra were acquired for 2,500 transients and a total acquisition time of 3 hours. ^1^H spectra were acquired with a 90° pulse and 3-second relaxation delay and 256 transients for a total acquisition time of 25 minutes. Spectra were quantified with ACD/Spec Manager version 9.15 software (Advanced Chemistry Development). Peak integrals obtained by deconvolution were normalized to an external standard (100 mM trimethylsilyl propionate [TSP] [Sigma-Aldrich] for ^13^C spectra and 10 mM methylene diphosphoric acid [MDPA] [Sigma-Aldrich] for ^31^P spectra) and to cell number and corrected for saturation effects by using data obtained from fully relaxed spectra of cell extracts as described above. In addition, studies were performed with choline concentration at the normal concentration in human plasma. For this, cells were grown with a 10 μM choline concentration in the medium, and cell extracts and MRS experiments were performed as above.

### Microarray analysis of gene expression

Total cellular RNA was isolated from approximately 1 × 10^7 ^cells after 48 hours of treatment with 17-AAG or vehicle (dimethyl sulfoxide) by using the RNeasy Mini Kit (Qiagen, Valencia, CA, USA) in accordance with the instructions of the manufacturer. RNA quality was determined by Bioanalyzer (Agilent Technologies, Santa Clara, CA, USA), and RNA integrity number (RIN) values of at least 8.0 were considered acceptable (most values were 9.5 or higher). Microarray hybridization was performed at the UCSF Genomics Core Laboratories (UCSF/Gladstone, San Francisco, CA, USA) by using the Human Gene 1.0 ST. Human Gene 1.0ST was analyzed by fluorescence detection by using the Agilent GeneArray Scanner (Agilent Technologies). Data acquisition was performed with Micro Array Suite 5.0 software (Affymetrix, Santa Clara, CA, USA). Microarray experiments were performed with four repeats of each condition. Microarray data are available through the ArrayExpress public repository [EMBL:E-MTAB-339] in compliance with the standards of the Microarray Data Gene Expression Society.

### Reverse transcription-polymerase chain reaction analysis of gene expression

An RNeasy Mini Kit (Qiagen) was used to extract total cellular RNA from samples. Total RNA concentration of samples was determined by using an ND1000 Fluorospectrometer (NanoDrop Technologies, Wilmington, DE, USA). The QuantiTect Reverse Transcription kit (Qiagen) was used to perform reverse transcription. Reverse transcription-polymerase chain reaction (RT-PCR) was performed on the resulting cDNA on a Taqman 7900 (Applied Biosystems, Carlsbad, CA, USA). Expressions of ChoKα, ChoKβ, and SLC44A1 were examined with Assays-on-Demand (Applied Biosystems) and normalized to the expression of the 18S ribosomal subunit (Integrated DNA Technologies, Coralville, IA, USA).

### Choline kinase activity assay

ChoK activity was measured in cell extracts by ^1^H MRS as previously described [40]. In brief, approximately 1 × 10^7 ^cells were resuspended in 500 μL of lysis buffer containing 100 mM Tris (pH 8.0), 1 mM ethylenediaminetetraacetic acid (EDTA), and 10 mM dithiothreitol (DTT) (all chemicals from Sigma-Aldrich). Lysate was repeatedly passaged through a fine-tipped needle (27.5-gauge) for homogenization and was sonicated 10 × 1 s at 20 kHz for membrane solubilization. The homogenate was centrifuged at 16,000 rpm for 30 minutes at 4°C, and 100 μL of reaction buffer containing 100 mM Tris (pH 8.0), 60 mM choline chloride, 120 mM ATP, and 120 mM magnesium chloride (all chemicals from Sigma-Aldrich) at the final concentration were added to the lysate supernatant. MRS experiments were performed at 25°C on a 600-MHz INOVA spectrometer (Varian). The conversion of choline to PC was measured in an array of ^1^H spectra over a 1-hour period. ^1^H spectra were obtained with a 90° pulse and 3-second relaxation delay. Choline and PC concentrations were determined as peak areas, and ChoK activity was determined by linear regression analysis of points in the linear portion of the curve representing the time course of PC formation.

### CTP:PC cytidylyltransferase activity assay

This assay was based on the method outlined by Vance and colleagues [41] in 1981 but with substantial modifications for compatibility with MRS. In brief, 2 × 10^7 ^cells were resuspended in 540 μL of lysis buffer containing 50 mM HEPES (pH 7.0) (Sigma-Aldrich), 5 mM EDTA (Sigma-Aldrich), 5 mM EGTA (ethylene glycol tetraacetic acid) (Sigma-Aldrich), 5.5 mM sodium bisulfite (Sigma-Aldrich), and 1 μL/mL protease inhibitor cocktail (Calbiochem). Lysate was repeatedly passaged through a fine-tipped needle (27.5-gauge) for homogenization and was sonicated 10 × 1 s at 20 kHz for membrane solubilization. The homogenate was centrifuged at 16,000 rpm for 30 minutes at 4°C. The cytidylyltransferase activity of the supernatant fraction was measured immediately after the addition of 60 μL of reaction mixture (final concentrations: 50 mM Tris-HCl [pH 8.0], 10 mM cytidine triphosphate [CTP], 5 mM PC, 5 mM DTT, and 25 mM MgCl_2_) (all chemicals from Sigma-Aldrich). MRS experiments were performed at 33°C on the 600-MHz INOVA spectrometer (Varian). Proton-decoupled (Waltz 16) ^31^P MR spectra were obtained with 30° pulse and 2.6-second repetition time. PC and CDP-choline concentrations were determined from peak areas. Cytidylyltransferase activity was determined by linear regression analysis of points in the linear portion of the curve representing the time course of CDP-choline formation.

### Phospholipase C activity assay

PtdCho-specific PLC activity was determined in cell lysates by using the EnzChek Direct Phospholipase C Assay (Invitrogen Corporation, Carlsbad, CA, USA). The assay measures PLC activity by addition of a proprietary substrate (glycero-phosphoethanolamine with a dye-labeled sn-2 acyl chain), which is cleaved by PtdCho-specific PLC. The cleavage releases the dye-labeled diacylglycerol, which produces a positive fluorescence signal that can be measured. Fluorescence (485 nm excitation and 535 nm emission) was measured by a SpectroFluor Plus spectrofluorometer (Tecan).

### Phospholipase A1/A2 activity assay

The activity of PtdCho-specific PLA1 and PLA2 enzymes was determined in cell lysates by using the EnzCheck Phospholipase A_1 _and Phospholipase A_2 _Assay kits (Invitrogen Corporation). The assay measures PLA1 activity by the addition of a proprietary substrate (dye-labeled glycerophosphoethanolamines with BODIPY FL dye-labeled acyl chain at the *sn*-1 position and dinitrophenyl quencher-modified head group). The *sn*-1 ester linkage of the substrate is cleaved by PLA1, which releases the dye-labeled acyl chain and produces a positive fluorescence signal that can be measured. The PLA2 activity assay measures the *sn-*2 ester link hydrolysis of the proprietary substrate 1-O-(6-BODIPY 558/568-aminohexyl)-2-BODIPY FL C5-Sn-glycero-3-phosphocholine by PLA2 enzyme, which releases the dye-labeled portion and produces a positive fluorescent signal that can be measured. Fluorescence (505 nm excitation and 515 nm emission) was measured by a SpectroFluor Plus spectrofluorometer (Tecan).

### Statistical analysis

The two-tailed Student *t *test was used to determine the statistical significance of the results, and a *P *value of not more than 0.05 was considered significant. All results are expressed as mean ± standard deviation.

## Results

### 17-AAG treatment depletes Hsp90 client proteins and inhibits cell proliferation

**Figure 2:**
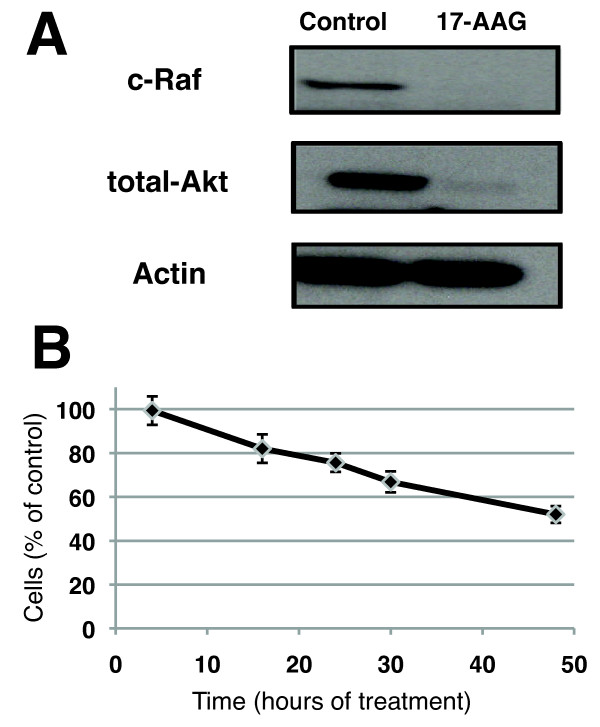
**Effect of 17-AAG on cell proliferation and Hsp90 client protein levels**. **(a) **Western blot analysis showing depletion of Hsp90 client proteins total-Akt and c-Raf following 17-AAG treatment of MCF-7 cells for 48 hours. Actin was used as a loading control. **(b) **Results of WST-1 assay showing 17-AAG effects on MCF-7 cell proliferation over a 48-hour treatment period. 17-AAG, 17-allyamino-17-demethoxygeldanamycin; Hsp90, heat shock protein 90.

Because our studies involved investigations of cells grown in flasks or on beads, we first confirmed that the proliferation rate of the cells was unaffected by the culture conditions. MCF-7 cell cultures in 75-cm^2 ^flasks had a doubling time of 29.5 ± 3.7 hours (*n *= 4) and this was comparable to a doubling time of 32.8 ± 2.7 hours for MCF-7 cells seeded on microcarrier beads (*n *= 4, *P *= 0.20). Of note, the doubling time of MCF-7 cells provided by the American Type Culture Collection (from which the cells were obtained) is 29 hours and is consistent with our findings. MCF-7 cells were treated for 48 hours with 3 μM 17-AAG. This led to the depletion of the known Hsp90 client proteins total-Akt and c-Raf (Figure 2a) and was associated with a decrease to 52% ± 3% in the number of cells as compared with control (Figure 2b). To further investigate the effect of 17-AAG treatment, FACS cell cycle analysis was performed after treatment for 48 hours. The results showed that the inhibitory effect of 17-AAG on cell proliferation is largely due to cell cycle arrest in the G_2_/M phase. The fraction of cells in the G_2 _phase increased from 8.4% ± 4.2% in control cells to 44.7% ± 5.2% in 17-AAG-treated cells (*n *= 3, *P *= 0.0008). The fraction of cells in the S phase decreased from 31.2% ± 2.3% in control cells to 4.6% ± 3.1% in 17-AAG-treated cells (*n *= 3, *P *= 0.0005). The fraction of cells in G_1 _phase was not significantly altered by 17-AAG treatment, and values for this fraction were 61% ± 5% in control cells and 51% ± 7% in 17-AAG-treated cells (*n *= 3, *P *= 0.12). Changes in relative cell size with 17-AAG treatment were also assessed. Whereas there was an increase in the size of cells in the G_1 _and S phases with 17-AAG treatment, there was a decrease in the size of cells in the G_2_/M phase population. Thus, given the entire cell population, the average cell size was not significantly altered, and 17-AAG-treated cells were 104% ± 4% the size of control cells (*n *= 3, *P *= 0.26). Consistent with this finding, average protein per cell was not significantly altered with 17-AAG treatment, and these values were 2.76 ± 0.05 mg/10^7 ^cells in control cells and 2.88 ± 0.25 mg/10^7 ^cells in 17-AAG-treated cells (*n *= 3, *P *= 0.12).

### 17-AAG treatment leads to increased PC levels and increased *de novo *PC synthesis

**Figure 3:**
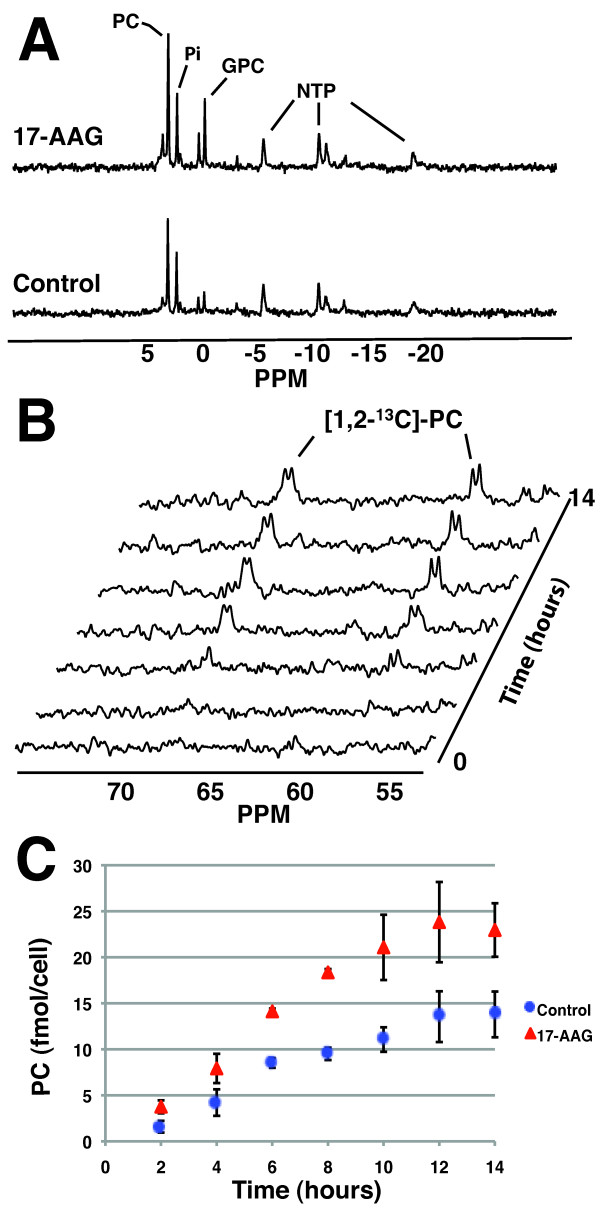
**Magnetic resonance spectroscopy detection of increased total and *de novo *PC in live, perfused cells following 17-AAG treatment**. **(a) **^31^P spectra of live, perfused MCF-7 cells illustrating an increase in PC after 48 hours of 17-AAG treatment. **(b) **^13^C spectral array depicting the build-up of [1,2-^13^C]-PC in perfused MCF-7 cells over a 14-hour period. **(c) **Graph of build-up of *de novo *[1,2-^13^C]-PC in control (blue) and 17-AAG-treated (red) MCF-7 cells over 14 hours of exposure to [1,2-^13^C]-choline. The data represent an average of three repeats. 17-AAG, 17-allyamino-17-demethoxygeldanamycin; GPC, glycerophosphocholine; NTP, nucleoside triphosphate; PC, phosphocholine; Pi, inorganic phosphate; PPM, parts per million.

To assess the effects of 17-AAG treatment on choline metabolism in live cells, ^31^P and ^13^C MR spectra were acquired from MCF-7 cells grown on beads and perfused in a 10-mm NMR (nuclear magnetic resonance) tube inside the spectrometer. First, a ^31^P MR spectrum was acquired in order to determine the total PC pool in both control and 17-AAG-treated cells (Figure 3a). PC concentrations increased significantly to 180% ± 19% of control in MCF-7 cells treated for 48 hours (*n *= 3, *P *= 0.004). Control cells had 19.3 ± 3.4 fmol/cell, and 17-AAG-treated cells had 34.7 ± 2.8 fmol/cell. Additionally, the concentration of GPC increased significantly with 17-AAG treatment to 216% ± 56% of control, from 2.5 ± 1.0 fmol/cell in control cells to 5.5 ± 1.3 fmol/cell in 17-AAG-treated cells (*n *= 3, *P *= 0.04). After 17-AAG treatment, there was no significant change in the β-NTP levels, which were 104% ± 12% of control (*n *= 3, *P *= 0.77).

The metabolism of live MCF-7 cells was then probed in real time by replacing choline in the medium with only [1,2-^13^C]-choline and by using ^13^C MRS to monitor the build-up of PC over a 14-hour period (Figure 3b). The data curve obtained displays the build-up of ^13^C-labeled PC inside the cells (Figure 3c). The level of ^13^C-labeled PC after 14 hours of exposure to labeled substrate was significantly higher in the cells treated with 17-AAG and increased with treatment to 171% ± 39% of control, from 14.2 ± 2.5 fmol/cell to 24.3 ± 1.6 fmol/cell (*n *= 3, *P *= 0.007). Relative to the total PC concentrations determined from the ^31^P spectrum, 75% ± 17% of the PC pool was ^13^C-labeled in control cells and 71% ± 10% of the pool was labeled in 17-AAG-treated cells after 14 hours of labeling, with no significant difference in percentage of labeling between control and treated cells (*n *= 3, *P *= 0.75).

At the end of the experiment, cells were extracted directly from the beads in order to assess ^13^C-label incorporation into PtdCho (owing to its low mobility within the cell membrane, the lipid cannot be detected in live cells) [38,39]. After 14 hours, less than 10% of the PtdCho pool was ^13^C-labeled and no [1,2-^13^C]-GPC peak was observed in either the control or treated samples. We thus concluded that the build-up of [1,2-^13^C]-PC observed over the first 14 hours was due mainly to the *de novo *synthesis of PC from extracellular labeled choline rather than to PtdCho breakdown.

The initial rate of PC synthesis was linear over the first 8 hours and was quantified for both control and 17-AAG-treated cells by using linear regression (R = 0.935 for control and R = 0.982 for treated). This analysis showed that the initial rate of PC synthesis increased significantly to 184% ± 7% of control with 17-AAG treatment, from 1.2 ± 0.1 fmol/cell per hour in control cells to 2.3 ± 0.4 fmol/cell per hour in treated cells (*n *= 3, *P *= 0.003). This increase was comparable to the increase observed in the total PC pool (180% ± 19% of control), indicating that increased *de novo *synthesis of PC is the major cause of PC elevation following 17-AAG treatment.

### 17-AAG does not affect PtdCho levels but increases total and *de novo *GPC and intracellular choline levels

**Figure 4:**
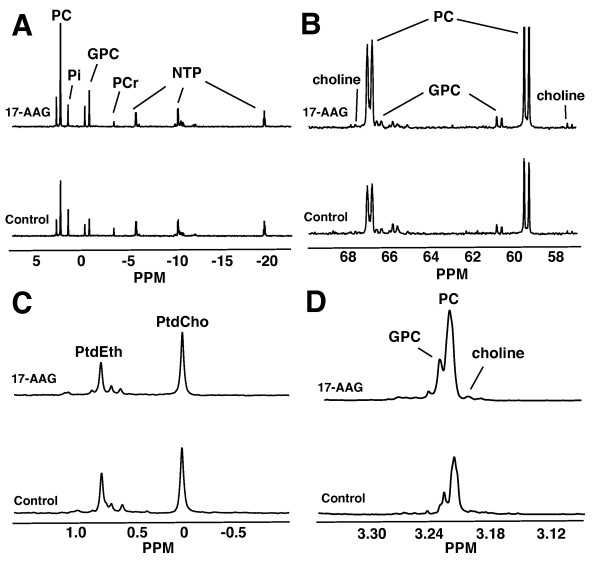
**Representative ^31^P, ^13^C, and ^1^H spectra of cell extracts**. Representative spectra of control (bottom) and 17-AAG-treated (top) MCF-7 cells illustrate increased PC, intracellular choline, and GPC levels and unchanged PtdCho levels. **(a) **^13^P spectra of the aqueous cell extract fraction depicting an increase in total PC and GPC levels after treatment. **(b) **^13^C spectra of the aqueous cell extract fraction depicting an increase in *de novo *synthesized PC, GPC, and intracellular choline. **(c) **^31^P spectra of the lipid cell extract fraction depicting constant PtdCho with 17-AAG treatment. **(d) **^1^H spectra of the aqueous cell extract fraction depicting an increase in the intracellular choline, PC, and GPC concentrations. 17-AAG, 17-allyamino-17-demethoxygeldanamycin; GPC, glycerophosphocholine; NTP, nucleoside triphosphate; PC, phosphocholine; PCr, phosphocreatine; Pi, inorganic phosphate; PPM, parts per million; PtdCho, phosphatidylcholine; PtdEth, phosphatidylethanol.

^1^H, ^31^P, and ^13^C MRS were used to quantify cellular metabolites in cell extracts following exposure to [1,2-^13^C]-choline for 48 hours in order to further confirm the observations made in live cells and to monitor *de novo *synthesis of PtdCho and GPC. Data from the extracts confirmed the findings from live cells: after 48 hours of treatment with 17-AAG, the total PC levels increased to 181% ± 10% relative to control, from 19.6 ± 1.2 to 35.4 ± 0.2 fmol/cell (*n *= 3, *P *= 0.001) (Figure 4a), and the *de novo *[1,2-^13^C]-PC levels increased significantly to 165% ± 10% of control, from 18.5 ± 1.6 fmol/cell to 30.6 ± 2.7 fmol/cell (*n *= 3, *P *= 0.006) (Figure 4b). Thus, after 48 hours of ^13^C labeling, the PC pool was essentially fully ^13^C-labeled, with 94% ± 3% of the pool labeled in control cells and 86% ± 11% of the pool labeled in the treated cells and no significant difference in the percentage of pool labeled between control and treated cells (*n *= 3, *P *= 0.20).

The concentration of total PtdCho was not changed significantly by 17-AAG treatment, with 25.2 ± 4.1 fmol/cell in control cells and 26.3 ± 4.0 fmol/cell in 17-AAG-treated cells (*n *= 3, *P *= 0.75) (Figure 4c). *De novo *synthesized [1,2-^13^C]-PtdCho levels were not significantly altered either. [1,2-^13^C]-PtdCho levels in 17-AAG-treated cells were 88% ± 11% of control, with 11.8 ± 2.6 fmol/cell in control cells and 10.3 ± 0.9 fmol/cell in 17-AAG-treated cells (*n *= 3, *P *= 0.44). After 48 hours, there was 46% ± 3% incorporation of ^13^C label into the total PtdCho pool in control cells and there was 40% ± 4% incorporation into the total pool in 17-AAG-treated cells, with no significant difference in the percentage of total PtdCho pool labeled between control and treated cells (*n *= 3, *P *= 0.06).

As in live cells, GPC levels increased significantly to 176% ± 38% relative to control (*n *= 3, *P *= 0.03). Control cells had 4.4 ± 0.9 fmol/cell, and 17-AAG-treated cells had 7.7 ± 1.3 fmol/cell (Figure 4a). *De novo *GPC levels also significantly increased with 17-AAG treatment to 149% ± 15% of control, from 1.9 ± 0.1 to 2.8 ± 0.3 fmol/cell (*n *= 3, *P *= 0.01) (Figure 4b). Of note, the difference between GPC levels determined in intact cells and in extracts was not significantly different (*P *> 0.05). After 48 hours, there was 44% ± 11% incorporation of ^13^C label into the total GPC pool in control cells and there was 37% ± 5% incorporation into the total pool in 17-AAG-treated cells, with no significant difference in the percentage of total GPC pool labeled between control and treated cells (*n *= 3, *P *= 0.36).

The concentration of total intracellular choline was determined by quantification of the ^1^H spectra (Figure 4d). Intracellular choline levels were significantly increased following 17-AAG treatment to 266% ± 18% of control, from 0.5 ± 0.1 fmol/cell in control cells to 1.3 ± 0.4 fmol/cell in treated cells (*n *= 3, *P *= 0.05). Consistent with these findings, intracellular [1,2-^13^C]-choline (as detected by ^13^C MRS; Figure 4b) was also increased in treated cells to 220% ± 27% of control, from 0.6 ± 0.1 fmol/cell in control cells to 1.2 ± 0.3 fmol/cell in treated cells (*n *= 3, *P *= 0.05). Thus, after incubation with medium containing [1,2-^13^C]-choline for 48 hours, the intracellular choline pool was essentially fully ^13^C-labeled. There was 116% ± 53% ^13^C enrichment of the intracellular choline pool in control cells and there was 95% ± 38% ^13^C enrichment in treated cells, with no significant difference in percentage of enrichment between control and treated cells (*n *= 3, *P *= 0.6).

### 17-AAG causes modulation in gene expression and activity of enzymes in the choline phosolipid metabolic pathway

In an effort to interpret the metabolic findings, microarray analysis was performed to determine the changes in gene expression associated with Hsp90 inhibition that could explain the alterations in choline metabolism observed following 17-AAG treatment. Table 1 lists the microarray data for enzymes involved in choline phospholipid metabolism.

**Table 1:** Summary of microarray data for enzymes associated with choline metabolism

Enzyme type	Gene symbol	Gene title	Percentage control	FDR
Choline transport	*SLC5A7*	Solute carrier family 5 (choline transporter), member 7	101	0.91
	*SLC44A1*	Solute carrier family 44, member 1	154^a^	2.10 × 10^-6^
	*SLC22A1*	Solute carrier family 22 (organic cation transporter), member 1	99	0.97
	*SLC22A2*	Solute carrier family 22 (organic cation transporter), member 2	97	0.81
Choline kinase	*CHKA*	Choline kinase alpha	109	0.2
	*CHKB*	Choline kinase beta	102	0.86
CTP:PC cytidylyltransferase	*PCYT1A*	Phosphate cytidylyltransferase 1, choline, alpha	101	0.92
	*PCYT1B*	Phosphate cytidylyltransferase 1, choline, beta	109	0.35
Diacylglycerol choline-phosphotransferase	*CHPT1*	Choline phosphotransferase 1	146^a^	2.4 × 10^-4^
Phospholipase A	*PLA1A*	Phospholipase A1 member A	121^a^	0.05
	*PLA2G1B*	Phospholipase A2, group IB (pancreas)	105	0.67
	*PLA2G2A*	Phospholipase A2, group IIA (platelets, synovial fluid)	101	0.96
	*PLA2G2C*	Phospholipase A2, group IIC	104	0.83
	*PLA2G2D*	Phospholipase A2, group IID	98	0.83
	*PLA2G2E*	Phospholipase A2, group IIE	98	0.91
	*PLA2G3*	Phospholipase A2, group III	99	0.18
	*PLA2G4C*	Phospholipase A2, group IVC (cytosolic, calcium-independent)	75^a^	0.0043
	*PLA2G4E*	Phospholipase A2, group IVE	101	0.95
	*PLA2G4F*	Phospholipase A2, group IVF	82^a^	0.018
	*PLA2G5*	Phospholipase A2, group V	97	0.87
	*PLA2G6*	Phospholipase A2, group VI (cytosolic, calcium-independent)	129	0.0067
	*PLA2G7*	Phospholipase A2, group VII	112	0.3
	*PLA2G10*	Phospholipase A2, group X	712^a^	1.60 × 10^-8^
	*PLA2G12A*	Phospholipase A2, group XIIA	87^a^	0.03
	*PLA2G12B*	Phospholipase A2, group XIIB	106	0.51
	*PLA2G15*	Phospholipase A2, group XV	139	0.00043
	*PLA2G16*	Phospholipase A2, group XVI	131	0.0021
Phospholipase	*PLD1*	Phospholipase D1, phosphatidylcholine-specific	137^a^	0.0038
	*PLD2*	Phospholipase D2	106	0.68
	*PLD3*	phospholipase D family, member 3	179^a^	7.80 × 10^-7^
	*PLD4*	Phospholipase D family, member 4	106	0.68
	*PLD5*	Phospholipase D family, member 5	116	0.1
	*PLD6*	Phospholipase D family, member 6	82^a^	0.0036
Lysophospholipase	*LYPLA1*	Lysophospholipase I	90	0.13
	*LYPLAL1*	Lysophospholipase-like 1	88	0.18
	*LYPLA2*	Lysophospholipase II	90	0.14
Glycero phosphocholin phosphodiesterase	*GDPD1*	Glycerophosphodiester phosphodiesterase domain containing 1	106	0.64
	*GDPD2*	Glycerophosphodiester phosphodiesterase domain containing 2	228^a^	1.2 × 10^-6^
	*GDPD3*	Glycerophosphodiester phosphodiesterase domain containing 3	142^a^	2.6 × 10^-7^
	*GDPD5*	Glycerophosphodiester phosphodiesterase domain containing 5	116	0.18
	*GDE1*	Glycerophosphodiester phosphodiesterase 1	112	0.062

^a^These enzymes had a statistically significant (false discovery rate [FDR] less than or equal to 0.05) modulation in expression with 17-allyamino-17-demethoxygeldanamycin (17-AAG) treatment.

### Choline transporters

Microarray analysis did not show significant changes in the gene expression of choline transporters SLC5A7, SLC22A1, or SLC22A2 but did show a significant increase in the mRNA expression of the choline transporter SLC44A1. RT-PCR confirmed the microarray data, with a significant increase in SLC44A1 mRNA levels to 169% ± 33% of control (*n *= 4, *P *= 0.04).

### Choline kinase

Microarray analysis did not show any significant change in the expression of ChoK α or β mRNA levels. RT-PCR was also performed to confirm the microarray data and did not show a significant change in ChoKα or ChoKβ mRNA levels (*n *= 4, *P *= 0.2). Finally, the cellular activity of ChoK remained essentially unchanged at 104% ± 23% of control; control cell extracts incubated with choline produced 98 ± 15 fmol PC/cell per hour, and 17-AAG-treated cells produced 102 ± 37 fmol PC/cell per hour (*n *= 4, *P *= 0.51).

### CTP:PC cytidylyltransferase

Microarray analysis did not show any significant changes in gene expression of the CCT isoenzymes. The activity of CCT also remained unchanged with 17-AAG treatment at 107% ± 21% of control; control cell extracts incubated with PC produced 18.2 ± 5.3 fmol CDP-choline/cell per hour, and 17-AAG-treated cells produced 19.4 ± 4.3 fmol CDP-choline/cell per hour (*n *= 4, *P *= 0.74).

### Phospholipases

Genes coding for PtdCho-specific PLC isoenzymes have not yet been identified, preventing gene expression analysis; however, PtdCho-specific PLC activity was assessed. The PtdCho-specific PLC activity assay performed on cell lysates showed a significant decrease in PLC activity with 17-AAG treatment to 66% ± 10% of control (*n *= 3, *P *= 0.02). Although there are many PLA isoenzymes, expression of PLA2G6, PLA2G10, PLA2G15, and PLA2G16 (all of which have activity on PtdCho) increased. Furthermore, the PtdCho-specific PLA1 and PLA2 enzyme activity assays did not find a significant increase in PLA1 activity but did show a significant increase in PLA2 activity to 122% ± 5% of control (*n *= 3, *P *= 0.02). Finally, microarray data reported an increase in PtdCho-specific PLD1 expression to 137% of control. There was also an increase in mRNA levels of the GDPD isoenzymes GDPD2 and GDPD3 in the microarray; however, neither of these forms is known to be specific for GPC. No significant changes were found in the microarray for any of the lysophospholipase isoenzymes.

### 17-AAG leads to an increase in PC levels at physiological choline concentration

Choline is transported into the cell by several choline transporters, each with different affinities for choline and differing activities over a range of choline concentrations [34,35]. Therefore, the overall rate of transport of choline into the cell may depend on the extracellular choline concentration. Since changes in expression of only one transporter were observed, we questioned whether 17-AAG would have the same effect on PC levels when extracellular choline concentrations are near physiological levels (more closely mimicking *in vivo *conditions). To address this question, MCF-7 cells were cultured in medium containing 10 μM choline (the concentration in human plasma) during the 48-hour treatment period [42,43]. ^31^P and ^1^H MR spectra showed an increase in PC levels to 202% ± 35% of control with 17-AAG treatment, from 17.0 ± 3.0 fmol/cell in control cells to 34.3 ± 4.0 fmol/cell in treated cells (*n *= 3, *P *= 0.005). There was also a significant increase in GPC levels to 214% ± 30% of control with 17-AAG treatment, from 3.7 ± 0.3 fmol/cell in control cells to 8.0 ± 1.7 fmol/cell in treated cells (*n *= 3, *P *= 0.04). Finally, intracellular choline levels were elevated to 194% ± 81% of control with treatment, from 1.1 ± 0.5 fmol/cell in control cells to 2.1 ± 0.8 fmol/cell in 17-AAG-treated cells, although this change did not reach statistical significance (*n *= 3, *P *= 0.15). Thus, the effect of 17-AAG on choline-containing metabolite levels was comparable (within experimental error) in cells cultured at physiological choline concentrations and cells cultured with 56 μM choline.

## Discussion

In our study, we examined the effects of Hsp90 inhibition on choline metabolism by treating MCF-7 breast cancer cells with the clinically relevant Hsp90 inhibitor, 17-AAG. We found that 17-AAG treatment of MCF-7 cells results in increases in intracellular choline, PC, and GPC concentrations at both normal cell culture and physiological extracellular choline concentrations. We also found that the most significant effect of 17-AAG on the enzymes associated with choline metabolism was an upregulation in expression of choline transporter SLC44A1 and PLA2.

In mammalian cells, choline is actively transported into the cell by various organic cation transporters [34,35]. The four main proteins responsible for choline transport in human cells are SLC5A7 (high-affinity choline transporter), SLC44A1 (intermediate-affinity choline transporter-like protein), and SLC22A1 and SLC22A2 (polyspecific organic-cation transporters with a very low affinity for choline). SLC44A1 is ubiquitously expressed in human tissues, implying that it is involved in choline metabolism for a broad purpose, such as phospholipid synthesis [44-47]. In MCF-7 cells, SLC44A1 is the most commonly expressed choline transporter (and mRNA expression levels are 2- to 3-fold higher than SLC22A1/2 and more than 30-fold higher than SLC5A7 [16]) and is localized on both the plasma and mitochondrial membranes [48]. In our study, 17-AAG did not cause significant changes in SLC5A7, SLC22A1, or SLC22A2 but did significantly increase expression of SLC44A1. Choline transport has been shown to be the rate-limiting step in the two-step formation of PC in breast cancer cell lines, including MCF-7 [49]. Since transport of choline into the cells is slower than phosphorylation of choline, an increase in choline transport would result in an increase in the synthesis of PC. Indeed, in our study, no significant changes in ChoK expression or activity were observed following 17-AAG treatment, ruling out the contribution of ChoK to the increased PC pool. The overexpression of choline transporter SLC44A1 is thus the most likely explanation for an increase in choline transport into the cell, and this would result in increased intracellular choline and *de novo *synthesis of PC following 17-AAG treatment.

The link between Hsp90 inhibition and increased SLC44A1 expression remains to be determined. Hsp90 is a chaperone protein with an extremely wide range of client proteins, including those with critical roles in signal transduction, cellular trafficking, chromatin remodeling, cell growth, differentiation, and reproduction [50]. With such a global effect on protein and gene expression, the metabolic changes observed could be due to a wide variety of causes. Studies on SLC44A1 are limited, with some indications that Sp1 or glucocorticoid receptor or both may play a role in its transcription [51-54]. However, neither of these transcription factors likely mediates the effect observed here, since both were reported to require active Hsp90 for their function and since treatment with the 17-AAG analogue geldanamycin abrogates their transcriptional activity [53,55]. Further studies are necessary to link the effect of Hsp90 to the regulation of choline transport.

Aside from the *de novo *synthesis of PC, two other potential mechanisms could contribute to PC accumulation: a decrease in the further metabolism of PC via CCT and an increase in the breakdown of PtdCho. We found no significant change in CCT expression or activity. Although CCT activity can be modulated by relocalization [56] or by changes in substrate concentration, the biological assay findings are consistent with the MRS data, in which there was no modulation in total PtdCho levels. This result is also in line with the fact that CCT is considered the overall rate-limiting step in the synthesis of PtdCho [36]. With regard to the breakdown of PtdCho, the expression of PLD increased to 137% following treatment. PLD could generate additional intracellular choline and possibly contribute to PC synthesis. However, the fact that essentially the entire intracellular choline pool was labeled following 48 hours of exposure to labeled choline makes this contribution unlikely. The activity of PLC, which could directly generate PC, dropped following 17-AAG treatment to 66% ± 10% of control. Thus, although modulation of the phospholipases could theoretically have a contribution to the increase in PC, the live cell and extract MRS data showing increases in total PC and *de novo *PC synthesis to the same extent, along with mRNA expression showing increased SLC44A1 expression to a similar degree, favor an increase in choline transport as the primary mechanism for increased intracellular choline and PC.

Regarding the observed increase in total and *de novo *GPC following 17-AAG treatment, we observed an increase in the activity of the PtdCho-specific PLA2 and microarray data showed an increase in five of the PLA2 isoenzymes. Thus, the increase in GPC is likely due to increased PtdCho breakdown in treated cells. Activation of PLA2 was also observed in response to other types of therapy [33]. However, we did not observe a significant change in total PtdCho levels. One possible explanation could be a lack of sensitivity: the concentration of PtdCho (25 ± 4 fmol/cell in control cells) is about five times greater than the concentration of GPC (4 ± 1 fmol/cell in control cells), and the change in GPC concentration from control to treated cells (an increase of 3.3 fmol/cell) is smaller than the standard deviation in the total PtdCho concentration. It is thus possible that our methods are not sensitive enough to detect such a small change. Alternatively, it is possible that inhibition of PLC (17-AAG caused PLC activity to decrease to 66% ± 10% of control) functions to counteract the elevated breakdown of PtdCho by PLA2, thereby maintaining a constant PtdCho pool.

Our findings are in agreement with those of the previous studies performed by Chung and colleagues [29] on human colon cancer cell lines, which reported an increase in PC concentrations in both cells and tumors when probed either with ^31^P or ^1^H MRS. Additionally, our results are in line with an increase in uptake of the PET tracer [^11^C]-choline reported in MCF-7 cells following Hsp90 inhibition [30]. This study, which measured choline uptake after cells were exposed to [^11^C]-choline for 30 minutes, supports our results that inhibition of Hsp90 causes an increase in the transport of choline. An increase in [^11^C]-choline uptake was also reported in human glioblastoma and human colon cancer cell lines, indicating that the mechanistic findings described here could potentially explain the metabolic effect of 17-AAG in other cancer cell types.

In contrast, Le and colleagues [57] reported a decrease in t-Cho levels of prostate cancer xenografts during the first 48 hours of treatment with 17-AAG, as measured by ^1^H MRSI. Since this was the first investigation of choline-containing metabolite levels following Hsp90 inhibition in a prostate cancer model, it is possible that this cancer type responds differently to Hsp90 inhibition. It is also possible that a longer treatment time is necessary to cause an increase in the concentration of PC. Indeed, the drop in PC reported by Le and colleagues was observed within 4 hours of treatment. Similarly, Liu and colleagues [58] reported that treatment with the Hsp90 inhibitor geldanamycin resulted in a drop in PC and a reduction in (methyl-^14^C) choline uptake within 1 hour of treatment. These rapid changes in choline uptake are unlikely to involve a change in protein expression, as was the case in our longer study.

## Conclusions

We have shown in our study, for the first time in a breast cancer model, that inhibition of Hsp90 results in a significant, MRS-detectable increase in intracellular choline, PC, and GPC. 17-AAG is currently being assessed in multiple clinical trials involving breast cancer patients, including patients who develop resistance to other therapies [2,5,59-61]. As Hsp90 inhibitors enter routine clinical use, the value of MRS as a noninvasive and localized imaging method to probe the effects of 17-AAG (and possibly other Hsp90 inhibitors) should thus be assessed further. ^1^H MRSI is an increasingly recognized imaging method that provides metabolic information that can help distinguish between benign and malignant lesions and inform on tumor response to treatment [62,63]. The t-Cho peak, which is detectable by ^1^H MRSI, is comprised of choline, PC, and GPC. The substantial increase in all three metabolites observed in this study would thus provide an easily detectable noninvasive biomarker of molecular drug response, which could enhance treatment monitoring and contribute to improved care for breast cancer patients.

## Abbreviations

17-AAG: 17-allyamino-17-demethoxygeldanamycin; CCT: CTP:phosphocholine cytidylyltransferase; CDP-choline: cytidine diphosphate-choline; ChoK: choline kinase; DMEM: Dulbecco's modified Eagle's medium; DTT: dithiothreitol; EDTA: ethylenediaminetetraacetic acid; FACS: fluorescence-activated cell sorting; GDPD: glycerophosphocholine phosphodiesterase; GPC: glycerophosphocholine; Hsp90: heat shock protein 90; MR: magnetic resonance; MRS: magnetic resonance spectroscopy; MRSI: magnetic resonance spectroscopic imaging; PC: phosphocholine; PET: positron emission tomography; PL: phospholipase A; PLC: phospholipase C; PLD: phospholipase D; PtdCho: phosphatidylcholine; RT-PCR: reverse transcription-polymerase chain reaction; t-Cho: total choline-containing metabolites.

## Competing interests

The authors declare that they have no competing interests.

## Authors' contributions

AHB acquired, analyzed, and interpreted experimental data; contributed to experimental conception and design; and drafted the manuscript. CSW acquired and analyzed microarray data, created the CTP:PC cytidylyltransferase activity assay, and contributed to experimental design. SMR conceived of the study, assisted with interpretation of data, and helped to draft the manuscript. All authors read and approved the final manuscript.
